# Anaerobic bacteria growth in the presence of cathelicidin LL-37 and selected ceragenins delivered as magnetic nanoparticles cargo

**DOI:** 10.1186/s12866-017-1075-6

**Published:** 2017-07-26

**Authors:** Bonita Durnaś, Ewelina Piktel, Marzena Wątek, Tomasz Wollny, Stanisław Góźdź, Jolanta Smok-Kalwat, Katarzyna Niemirowicz, Paul B. Savage, Robert Bucki

**Affiliations:** 1 Department of Microbiology and Immunology, The Faculty of Health Sciences of the Jan Kochanowski University in Kielce, Kielce, Poland; 2 Holy Cross Oncology Center of Kielce, Artwińskiego 3, Kielce, Poland; 3 Department of Microbiological and Nanobiomedical Engineering, Medical University of Białystok, Mickiewicza 2C, Białystok, Poland; 4 Department of Chemistry and Biochemistry, Brigham Young University, Provo, UT USA

**Keywords:** Anaerobic bacteria, Ceragenins, Cathelicidin, Magnetic nanoparticles

## Abstract

**Background:**

Cationic antibacterial peptides (CAPs) and synthetic molecules mimicking the amphiphilic structure of CAPs, such as ceragenins, are promising compounds for the development of new antimicrobials.

**Results:**

We tested the in vitro activity of ceragenins CSA-13 and CSA-131 against several anaerobic bacteria including *Bacteroides* spp. and *Clostridium difficile*. We compared results to the activity of cathelicidin LL-37, metronidazole and nanosystems developed by attachment of CSA-13 and CSA-131 to magnetic nanoparticles (MNPs). The antibacterial effect was tested using killing assay and modified CLSI broth microdilution assay. Ceragenins CSA-13 and CSA-131 displayed stronger bactericidal activity than LL-37 or metronidazole against all of the tested bacterial strains. Additionally CSA-131 revealed an enhanced ability to prevent the formation of *Bacteroides fragilis* and *Propionibacterium acnes* biofilms.

**Conclusions:**

These data confirmed that ceragenins display antimicrobial activity against a broad range of microorganisms including anaerobic bacteria and deserve further investigations as compounds serving to develop new treatment against anaerobic and mixed infections.

## Background

Anaerobic bacteria are part of the human microbiota, colonizing the skin and mucosal membranes of the oral cavity, gut, and genitourinary system. As commensals, they participate in numerous immunological and physiological processes, maintaining host homeostasis [1]. On the other hand, as opportunistic microorganisms, they can be responsible for a variety of endogenous infections in almost all body sites including the head and neck region, central nervous system, chest, abdomen, female genital tract, skin and soft tissues [2]. Additionally, they can also cause bacteremia, an infrequent occurrence associated with high mortality [3].

Many of these infections are polymicrobial (with aerobic and facultative bacteria) and occur when the skin or mucosal barriers are disrupted by surgery, tumors, ischemia, necrosis or trauma. In such conditions local tissue redox potentials are reduced and microorganisms can enter sites that were previously sterile. The most common anaerobes isolated from clinical samples are *Bacteroides* spp., *Clostridium* spp., *Prevotella* spp., and *Peptostreptococcus* spp. [4]. Certain anaerobic bacteria are involved in the pathogenesis of specific diseases. For example: *Porphyromonas gingivalis* and *Fusobacterium nucleatum* in periodontal diseases [5], *Gardnerella vaginalis*, *Mobiluncus *spp., *Atopobium vaginae* in bacterial vaginosis [6] *Clostridium difficile* in antibiotic-associated diarrhea and colitis [7], *Propionbacterium acnes* in acne vulgaris [8], and *Clostridium perfringens* in gas gangrene of the soft tissue and alimentary intoxication [9].

In addition to the use of antibiotics, such as metronidazole, clindamycin, cefoxitin, carbapenems or tigecycline, many anaerobic infections require the use of additional procedures for effective treatment. These include abscess drainage, debridement of necrotic tissue, decompression of infected spaces and removal of foreign bodies [10]. In recent years, many anaerobic bacteria show a tendency to develop antibiotic resistance. This is particularly true for clinical strains of the *Bacteroides* species. These bacteria exhibit increasing resistance to penicillin, cefoxitin, clindamycin, chloramphenicol, metronidazole and imipenem [11]**.** Moreover, some infections are difficult to treat due to recurrence (e.g. bacterial vaginosis, *Clostridium difficile* related diarrhea) or because of its chronic character (e.g., periodontitis). For these reasons novel strategies are required to treat infections involving anaerobic bacteria.

Natural CAPs such as human cathelicidin LL-37 display activity against a wide range of microorganisms. LL-37 is an amphipathic, helical peptide found in leukocytes as well as epithelial cells of the testis, skin, gastrointestinal and respiratory tract. In addition to its antimicrobial properties, LL-37 has several immunomodulatory functions including bacterial lipopolysaccharide (LPS) neutralization, chemotactic activity towards leukocytes, induction of angiogenesis, enhanced wound healing, and tissue regeneration [12]. Ceragenins (CSAs) are a family of lipid compounds that mimic the amphiphilic character of endogenous LL-37. As derivatives of bile acids they are resistant to digestion by proteases and can be easier and less costly to prepare during chemical synthesis. Ceragenins reproduce the broad-spectrum antibacterial activity of LL-37 against both Gram-positive and Gram-negative microorganisms including multi-resistant strains [13]. Similarly to LL-37, CSAs are active against planktonic microbes as well as established biofilms of both Gram-positive and Gram-negative bacteria [14]. In addition to robust activity, efficient penetration within infection sites is necessary for effective antimicrobial treatment. At sites of anaerobic infection, when abscesses or necrotic tissues are present, efficient drug concentrations are particularly difficult to obtain. The use of controlled drug delivery systems (DDS), such as magnetic nanoparticles (MNPs), could be a new promising solution to transport drugs to the place of action. The main advantages of MNPs are easy handling with the aid of magnetic field and low toxicity [15]. Moreover, it has been shown that MNPs display activity against certain aerobic microorganisms [16]. In the present study we assess for the first time the in vitro activity of ceragenins (CSA-13 and CSA-131) against several species of anaerobic bacteria and compare their activity to those of cathelicidin LL-37 and the conventional antibiotic metronidazole. We also investigate the activity of the mentioned substances attached to the surface of MNPs.

## Methods

### Bacterial strains and culture conditions

The following anaerobic bacterial strains were used: the laboratory strains *Bacteroides fragilis* (ATCC 25285) and *Propionibacterium acnes* (ATCC 11827) from American Type Culture Collection and 9 clinical strains isolated from patients hospitalized in the Holly Cross Cancer Centre in Kielce (Poland). These clinical isolates include the following organisms: Gram-negative - *Bacteroides thetaiotaomicron, Bacteroides stercoris, Prevotella melaninogenica, Prevotella oralis, Prevotella bivia, Prevotella disiens;* and Gram-positive *- Clostridium perfringens, Clostridium difficile, Peptostreptococcus* spp. All clinical strains were isolated from samples routinely cultured on Schaedler agar supplemented with 5% sheep blood (bioMérieux) in anaerobic conditions (5% CO_2_, 10% H_2_, and 85% N_2_, Anaerobic Work Station BUG BOX, JOUAN), identified by VITEK 2 Compact (bioMérieux) and stored in MAST CRYOBANK system (Mast Diagnostica) at −70 °C. The stored strains were grown on Schaedler agar supplemented with 5% sheep blood (bioMérieux) in anaerobic conditions. The characteristics of clinical strains is presented in Table 1.

**Table 1:** Characteristic of clinical isolates

Strain	Source of strains	Patient’s sex/age
*Bacterides thetaiotaomicron*	postoperative gynecological wound	F/66
*Bacteroides stercoris*	peritoneal fluid	F/81
*Prevotella melaninogenica*	postoperative wound in oral cavity region	F/80
*Prevotella oralis*	postoperative wound in oral cavity region	F/86
*Prevotella bivia*	postoperative wound in genitor-urinary tract	F/75
*Prevotella disiens*	postoperative gynecological wound	F/86
*Clostridium perfringens*	postoperative colon wound	M/51
*Clostridium difficile* producing A and B toxins	antibiotic-associated diarrhea	F/46
*Peptostreptococcus* spp.	breast abscessus	F/58

### Antimicrobial compounds and synthesis of tested nanosystems

Human cathelicidin LL-37 peptide was purchased from Polish Peptide Laboratory (Łódź, Poland). The ceragenins, CSA-13 and CSA-131, were synthesized as described previously [17]. Magnetic nanoparticles (MNP) were synthesized by Massart’s methods with modification based on co-precipitation of iron chloride salts under treatment by 25% ammonium hydroxide [18]. Immobilization of ceragenins and metronidazole on the surface MNPs was perfomed through the reaction of the antimicrobial compound with the magnetic nanoparticles’ aldehyde groups while functionalization by LL-37, were made via reaction of amidation. Briefly, bare MNPs were coated with an aminosilane layer and then glutaric dialdehyde was added. This process resulted in the formation of terminal aldehyde groups able to interact with ceragenins, metronidazole or ampicillin. Magnetic nanoparticles with amine or aldehyde groups were resuspended in dimethyl sulfoxide (DMSO) containing the antibacterial molecules. After functionalization, the precipitate was magnetically collected by an external magnetic field, washed and dried to powder. The physicochemical properties of the covalently attached antibacterial compounds including spectral (FT-IR), thermal (DSC, TGA) and morphological (TEM) analysis were characterized as previously described [19]. Total amount of molecules attached on MNPs surface were calculated based on results from weight loss in thermogravimetric analysis as well as total amount of amine group on the MNP surface.

### Antibacterial testing - killing assays

Killing assays were performed against: *Bacteroides fragilis* ATCC 25285, *Propionibacterium acnes* ATCC 11827 and a clinical isolate of *Clostridium difficile*. For the remainder of the clinical strains, MIC/MBC (minimum inhibitory concentration/minimum bactericidal concentration) were determined. In killing assay the efficiency of LL-37, CSA-13, CSA-131, metronidazole (MET), ampicillin (AM) and the tested nanosystems (ranged from 0.1 to 100 μg/ml) were evaluated in triplicate. Briefly, bacterial cells were cultured on Schaedler agar supplemented with 5% sheep blood to mid-log phase at 37 °C in anaerobic conditions, suspended in PBS (phosphate-buffered saline) to a final cell density 10^6^ CFU (colony forming unit)/ml per well (10^8^ CFU/ml for *Clostridium difficile*) and added to antimicrobials. When ready, microtiter plate was covered by gas permeable membrane and placed in an anaerobic chamber and incubated for 1 h at 37 °C. After incubation, the plates were transferred to ice, and suspensions were diluted serially from 1:10 to 1:1000 in PBS. Then, 10 μl aliquots from each well were spotted twice on plates with Schaedler agar supplemented with 5% sheep blood and cultured at 37 °C in anaerobic condition for 48 h (for *Propionibacterium acnes* 96 h). Following incubation, the numbers of CFUs were determined. Each experimental stage was performed with culture controls in anaerobic as well as in aerobic conditions.

### Antibacterial testing - MIC/MBC evaluation

To determine the minimum inhibitory concentrations (MICs), broth microdilution assays were performed. In our experiments, we modified the original CLSI (Clinical Laboratory Standards Institute) microdilution method (approved only for *Bacteroides fragilis* group) where the recommended medium is the Brucella broth supplemented with 1 μg/ml vitamin K1, 5 μg/ml hemin and 5% laked sheep blood [20]. Our intention was to use the comparable experimental conditions for all clinical strains and all tested compounds. We performed the initial MIC assessment for all tested compounds in the recommended by CLSI Brucella broth (Graso, Poland) with supplements: vitamin K1, hemin (BBL) and laked sheep blood (BioMaxima, Poland); than in the Brucella broth with vitamin K and hemin but without sheep blood. We selected two representative bacteria strains: for the first medium - laboratory *Bacteroides fragilis* ATCC 25285 strain, for the second medium – clinical strain *Prevotella oralis*. Additionally, we checked the ability of tested strains to growth in Brucella broth. Finaly, for the main part of performed experiment we used the Brucella broth without supplements and continue with this method for the *Bacteroides* and *Prevotella* strains as well as *Clostridium perfingens* and *Peptostreptococcus* spp.

The broth microdilution assay was performed in 96-well polypropylene round bottom microtiter plates. Briefly, serial two-fold dilutions of the tested antimicrobial agents were prepared in Brucella broth ranging from 256 μg/ml to 0.5 μg/ml. The inoculum of each isolate, prepared directly from fresh 18–24 h culture, were added to the wells to a final concentration of 5 × 10^5^ CFU/ml per well, then incubated in anaerobic conditions for 48 h at 37 °C. The MICs were determined visually as the lowest concentration of tested agents that inhibited the microbial growth. The MBCs values were assessed by plating each sample on Schaedler agar supplemented with 5% sheep blood and culturing at 37 °C in anaerobic condition for 48 h. Additional growth controls were performed in anaerobic as well as in aerobic conditions for each run.

### Activity against biofilm

Biofilms were grown in microtiter plates for 48 h at 37 °C in anaerobic conditions using Brucella broth with or without the antibacterial substances, ranging in concentration from from 1 to 100 μg/ml. After incubation, the content of each well were removed and each well was carefully washed with PBS to remove planktonic cells The plates were dried at room temperature and stained for 15 min using 150 μL of 0.1% crystal violet. Then, the excess stain was removed and the biofilms were rinsed with deionized water and the plates left to dry. To solubilize the crystal violet, 100 μl of 98% ethanol was added to each well. Then, 100 μl of ethanol was added and optical density (OD) was determined at a wavelength of 570 nm using an automatic Elisa microplate reader (BioTekInstrument). OD values indicate the amount of bacteria adhering to the surface and forming a biofilm [21].

### Statistical analysis

Killing assay measurements and biofilm assays were performed in triplicate. Experimental data were evaluated using the one-way analysis of variance (ANOVA) and Tukey HSD Post-hoc analyses. Statistical assessment was performed using Statistica 10 (StatSoft Inc., Tulsa, OK, USA). *P* < 0.05 was considered to be statistically significant.

## Results

### Ceragenins CSA-13 and CSA-131 exert antibacterial activity against anaerobic species

**Fig. 1:**
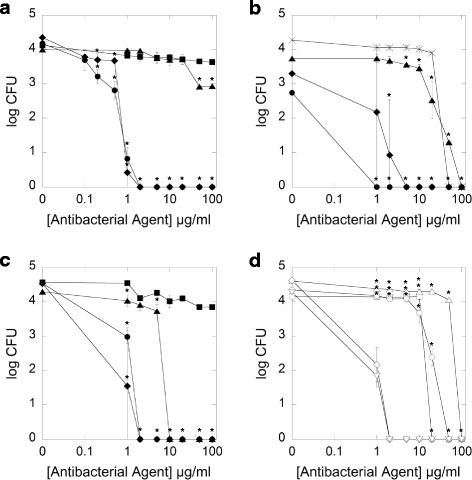
Antibacterial activity of metronidazole (*filled squares*), LL-37 peptide (*filled triangles*), and ceragenins: CSA-13 (*filled circles*) and CSA-131 (*filled diamonds*) against *Clostridium difficile* (panel **a**). Antibacterial activity of ampicillin (*cross*), LL-37 peptide (*filled triangles*), and ceragenins: CSA-13 (*filled circles*) and CSA-131 (*filled diamonds*) against *Propionibacterium acnes* (panel **b**). Antibacterial activity of metronidazole (*filled squares*), LL-37 peptide (*filled triangles*) and ceragenins: CSA-13 (*filled circles*), and CSA-131 (*filled diamonds*) against *Bacteroides fragilis* (panel **c**). Antibacterial activity of magnetic nanoparticles (MNPs) functionalized by metronidazole (MNP@MET; *empty squares*), MNPs functionalized by LL-37 peptide (MNP@LL37; *empty* *triangles*), MNPs functionalized by CSA-13 (MNP@CSA13; *empty* *circles*) and MNPs functionalized by CSA-131 (MNP@CSA131; *empty* *diamonds*) when compared to activity of unmodified nanoparticles (MNPs; *empty inverted* *triangles*) against *Bacteroides fragilis* (panel **d**). Error bars represent standard deviations from three measurements. *indicates statistically significant (*p* ≤ 0.05) activity of tested agents compared to metronidazole (panels **a** and **c**), ampicillin (panel **b**) and uncoated MNPs (panel **d**)

A killing assay was used as an initial screening method to assess the antibacterial activity of the tested compounds. Additionally, MIC/MBC values were measured to determine their clinical potential for anaerobic bacteria eradication. As shown in Fig. 1 (panels a, b, c), in the killing assay both tested ceragenins display a greater bactericidal activity than LL-37 and the antibiotics MET and AM. Bacterial growth inhibition occurred at 2 μg/ml of CSA-13 and CSA-131 against *C. difficile* (Fig. 1a) and *B. fragilis* (Fig. 1c), at 1 μg/ml of CSA-13 and 8 μg/ml of CSA-131 against *P. acnes* (Fig. 1b). In contrast, LL-37 (100 μg/ml) inhibits the growth of *P. acnes*. Interestingly at such high concentration cathelicidin LL-37 was unable to inhibit the growth of *C. difficile* (Fig. 1a, b). The bactericidal activity of LL-37 was only observed for *B. fragilis* (Fig. 1c), at a dose of 10 μg/ml.

In the initial MIC assessment performed in the medium recommended by CLSI - Brucella broth with vitamin K1, hemin and laked sheep, we observed inhibition of LL-37 and tested ceragenin killing properties (the MICs values for these compounds were in a range 128–256 mg/l). Similar MICs values were observed when Brucella broth containing hemin, vitamin K1 but lacking sheep blood was used. MICs values for metronidazole in the recommended and modified media were more comparable. These results in comparison with MIC values performed in Brucella broth without supplements are presented in Table 2. These initial results indicated that the supplements (probably hemin) inhibited the antimicrobial activity of tested molecules. It is worth mentioning that hemin is not a substance present in the human body, therefore its inhibitory effects on the tested compounds has no clinical implications. So, to avoid the inhibitory effect of supplements on the activity of LL-37 and tested ceragenins, Brucella broth without additives was used. Additionally to keep the same experimental conditions, we applied this medium for all tested antimicrobial compounds, including conventional antibiotic metroniazole.

**Table 2:** Minimal inhibitory concentration (MIC, mg/L) assessed for CSA-13, CSA-131, LL-37, MET and magnetic nanoparticles (MNP) including MNPs functionalized with tested antibacterial agents, assessed in different combination of media against two anaerobic bacteria strains

		MIC [mg/L]								
Strain	Medium used for MIC evaluation	Met	MNP@ Met	LL-37	MNP@ LL-37	CSA-13	MNP@ CSA13	CSA 131	MNP@ CSA 131	MNP
*Bacteroides fragilis* ATCC 25285	Brucella broth supplemented with 1 μg/ml vitamin K1, 5 μg/ml hemin and 5% laked sheep blood	1	2	>256	>256	>256	>256	128	128	>256
*Prevotella oralis*	Brucella broth supplemented with 1 μg/ml vitamin K1 and 5 μg/ml hemin	2	2	256	256	128	128	128	128	256

We next used our modified CLSI broth microdilution assay to evaluate the MIC/MBC values of LL-37, CSA-13, CSA-131 and metronidazole against reference *B. fragilis* ATCC 25285 and 9 clinical anaerobic strains (Table 3). Because in the first experimental setting we did not obtain the sufficient *Clostridium difficile* growth in Brucella broth, we excluded this microorganism from MIC/MBC evaluation. The antibacterial activity for this bacterium was assessed using killing assay. Compared to its results, the antibacterial effects were weaker, especially for LL-37. Among the tested compounds, CSA-13 displayed the highest antibacterial activity against all examined bacterial species. The small differences between MIC and MBC values indicate that the effect is bactericidal. Even though the differences in MIC are from 0.5 to 32 times higher for CSA-13 compared to MET, CSA-13 is definitely more efficient than LL-37*.* Among all three examined compounds, LL-37 had the highest MICs values for all strains: 128 mg/l for *Bacteroides* strains, 8–128 mg/l for *Prevotella* strains, 128 mg/l for *Clostridium perfringens* and 8 mg/l for *Peptostreptococcus* spp. Although, the differences between the results obtained from the killing assay and MIC/MBC evaluation are associated with different experimental setting: non-growth conditions versus the growth conditions.

**Table 3:** Minimal inhibitory concentration (MIC, mg/l) and minimal bactericidal concentration (MBC mg/l) of CSA-13, CSA-131, LL-37, MET and these agents functionalized on magnetic nanoparticles (MNP) and MNP against tested anaerobic strains (Brucella broth with no supplements – vitamin K1, hemin nor sheep blood)

	MIC/MBC [mg/l]								
Strain	Met	MNP@Met	LL-37	MNP@LL-37	CSA-13	MNP@CSA-13	CSA-131	MNP@CSA-131	MNP
*Bacteroides fragilis*	0.25/0.5	0.25/0.5	128/256	128/256	4/8	2/4	8/16	4/4	>256/>256
*Bacterides thetaiotaomicron*	0.25/0.5	0.25/0.5	128/128	128/128	8/8	2/2	8/8	16/16	>256/>256
*Bacteroides stercoris*	0.25/0.5	0.25/0.5	128/128	128/256	2/2	2/2	8/16	16/32	>256/>256
*Prevotella melaninogenica*	0.25/0.5	0.25/0.5	128/128	128/128	8/8	2/2	8/8	16/16	256/256
*Prevotella oralis*	1/1	1/1	8/8	16/32	0.5/0.5	1/1	4/4	16/16	256/256
*Prevotella bivia*	1/1	0.5/0.5	64/64	32/32	1/1	2/2	2/4	2/4	>256/>256
*Prevotella disiens*	0.125/0.125	0.125/0.125	16/16	16/16	1/1	1/1	2/2	4/4	>256/>256
*Clostridium perfringens*	1/1	2/2	128/256	64/128	1/1	1/1	4/4	16/16	>256/>256
*Peptostreptococcus spp.*	0.5/0.5	0.5/0.5	8/8	16/16	0.5/0.5	0.5/0.5	4/4	8/8	>256/>256

### CSA-131 decreases *Bacteroides fragilis* and *Propionibacterium acnes* biofilm formation

**Fig. 2:**
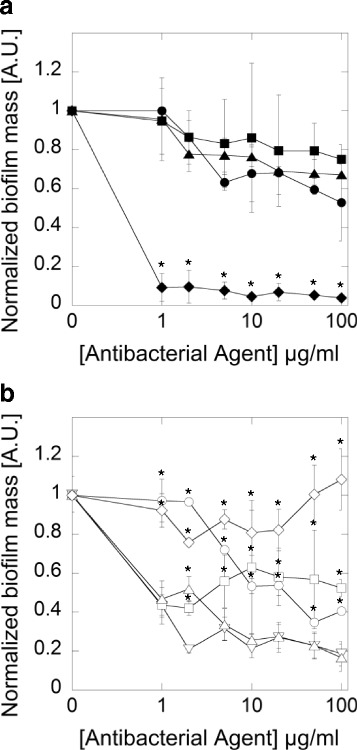
Decrease of *Bacteroides fragilis* biofilm formation induced by metronidazole (*filled squares*), LL-37 peptide (*filled triangles*), CSA-13 (*filled circles*) and CSA-131 (*filled diamonds*) (panel **a**) and MNPs functionalized by these compounds: MNP@MET (*empty squares*), MNP@LL-37 (*empty triangles*), MNP@CSA-13 (*empty circles*), MNP@CSA-131 (*empty diamonds*) and unfunctionalized MNPs (*empty inverted triangles*) (panel **b**). Error bars represent standard deviations from three measurements. *indicates statistically significant (*p* ≤ 0.05) activity of tested agents compared to metronidazole (panel **a**) and uncoated MNPs (panel **b**)

**Fig. 3:**
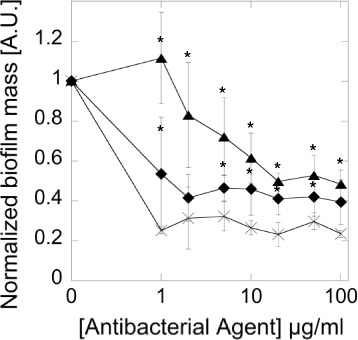
Decrease of *Propionibacterium acnes* biofilm formation induced by AM (*cross*), LL-37 peptide (*filled triangles*) and CSA-131 (*filled diamonds*). Error bars represent standard deviations from three measurements. *indicates statistically significant (*p* ≤ 0.05) activity of tested agents compared to ampicillin

To investigate the effects of ceragenins on biofilm formation we selected two strains: Gram-negative *Bacteroides fragilis* and Gram-positive *Propionibacterium acnes*. Ceragenin CSA-131 significantly inhibited biofilm formation by *B. fragilis* whereas the antibiofilm activities of CSA-13 and LL-37 were comparable to metronidazole (Fig. 2). The normalized biofilm mass formed by *B. fragilis* in the presence of CSA-131 at 1 μg/ml was lower when compared to the same dose of metronidazole. Because CSA-131 displayed higher anti-biofilm activity compared to CSA-13, we assessed CSA-131 effect on *Propionibacterium acnes* biofilm formation (Fig. 3). In this case the inhibitory effect of CSA-131 was better than that of LL-37 and comparable with the conventional antibiotic ampicillin, especially at low concentration.

### Immobilization of antibacterial agents LL-37, ceragenins and metronidazole onto the surface of magnetic nanoparticles changes bactericidal effect

Parallel experiments (killing assay, MIC/MBC evaluation, biofilm activity) were performed with CSA-13, CSA-131, LL-37 and metronidazole attached to MNPs (MNP@CSA-13, MNP@-131, MNP@LL-37, MNP@MET). Results of the killing assays with antibacterials functionalized on MNPs are shown in Fig. 1d. Surprisingly, MNPs without antimicrobials exerted the greatest antimicrobial effects against *B. fragilis.* However, this activity is very similar for MNP@CSA-131. In contrast, the MNP@MET, MNP@CSA-13 and MNP@LL-37 compounds had substantially lower activity. Comparing the tested compounds attached to MNPs to free compounds (Fig. 1a  to c) it is evident that ceragenins and LL-37 without nanoparticles were slightly more active than with nanoparticles. The opposite effect was observed for metronidazole.

Taking together results of the killing assay and MIC/MBC for *B. fragilis*, antibacterials functionalized on MNPs were less effective than antibacterials alone. The MIC values for MNPs against the reference *B. fragilis* strain were above 256 μg/ml. For nanoparticles with metronidazole, LL-37, CSA-13 and CSA-131 the MIC/MBC values were the same or slightly lower than in situations when antimicrobials were used alone. These unexpected results support the probable oxidative mechanisms of action of magnetic nanopatrticles via the interference with the transport during oxidation of nicotinamide adenine dinucleotide in bacteria [16]. Additional mechanism that might explain strong effect of bear MNPs potentially involves direct interaction of bacteria cells with the MNP surface that is stronger when MNPs are not modified.

Most antimicrobials attached to MNPs, even MNPs alone, exhibited enhanced antibiofilm activity (Fig. 2b). The only exception was CSA-131, which at doses above 20 μg/ml was shown to stimulate biofilm formation. Surprisingly, when CSA-131 was used alone, it exerted the strongest anti-biofilm activity among all tested compounds.

## Discussion

Slow growth rates, the ability to form biofilms, and increasing antibiotic resistance are three major challenges in the treatment of anaerobic bacterial infections. Furthermore, their fastidious nature, high nutritive requirements and susceptibility to oxygen make anaerobes difficult to isolate from clinical materials. Because of this, antibiotic treatment often relies heavily on empirical decision making [22]. Antibacterial peptides and ceragenins are promising compounds for the development of new antimicrobials. The effectiveness of CSAs against different aerobic bacteria, including multidrug-resistant strains, has previously been demonstrated [23, 24]. This study represents one of the first showing the activity of ceragenins against anaerobic microorganisms. The evaluation of antibacterial activity against anaerobs is complicated due to the technical difficulties of isolation, culture and susceptibility assessment. In a study by Oh et al*.*, the activity of the hybrid peptide cecropin-melittin analogues (CAMEL) towards various clinical anaerobic species including *B. fragilis, F. nucleaatum, Prevotella* spp., *Propionibacterium* spp. and *Peptostreptococcus* spp., was found to be equal or superior compare to those of metronidazole, cefoxitin, ciprofloxacin and chloramphenicol, although inferior to imipenem, clindamycin and piperacillin [25]. The antibacterial activity of peptides initially isolated from the skin of frog in general had good potency against Gram-positive anaerobic bacteria but poor against Gram-negative (with the exception of *Prevotella melaninogenica)* [26]. The SMAP-29 peptide, a member of the cathelicidin family, displayed activity against *Bacteroides fragilis, Clostridium perfringens* and *Clostridium difficile*. The mechanism of action leading to bacterial death is similar to aerobic bacteria, despite differences in the membrane lipid composition between these two groups of microorganisms [27]. However, more recent data have described the resistance of anaerobics such as *Porphyromonas gingivalis* to antimicrobial peptides resulting from strong proteolytic activity and a low affinity for positively charged peptides unique to its LPS composition [28].

In the current study we evaluated the in vitro activity of human cathelicidin LL-37 and the ceragenins CSA-13 and CSA-131 against several anaerobic bacteria associated with human infection. We assessed the activity of the tested compounds in two different experimental setting: non-growth conditions (PBS and 1 h incubation) using a killing assay and growth conditions (high nutrient medium and 48 h of incubation) using MIC/MBC evaluation. The results of these two experiments show that LL-37 has bactericidal activity against anaerobic bacteria, but because MIC/MBC values are high, this activity would be insufficient against growing cells. Ouhara et al. observed similar results when assessing the activity of LL-37 and other antibacterial peptides towards periodonto-pathogenic and cariogenic bacteria [29]. It is possible, that the differences observed between growth and non-growth conditions are greater for anaerobics compared to aerobic bacteria due to the slower anaerobics growth rates. In our study, activity of the tested compounds was variable against the different species and strains. Since the main mechanism of action for CAPs and CSAs involves charge driven destabilization of cell membrane lipid organization [30], it has been postulated that bacterial charge, which can be different even within a species, may influence susceptibility to cationic antibacterial peptides [29]. In our study, compared to metronidazole, MIC/MBC values for CSA-13 were slightly higher against Gram-negative (*Bacteroides* and *Prevotella* species) and the same against Gram-positive bacteria *(Clostrdium perfringens and Peptostreptococcus spp).* MIC/MBC values for CSA-131 was higher, and for LL-37, significantly higher than those of MET (for both Gram-positive and Gram-negative bacteria). Because we modified the original CLSI method, our results cannot be compared with MIC determined using standard protocol. However they can be used for comparing antibacterial activities of tested compounds in our experimental setting. Metronidazole is the drug of choice for the management and prophylaxis of anaerobic infections. It is highly active against Gram-negative anaerobic rods (e.g. *Bacteroides* species, fusobacteria) as well as Gram-positive such as clostridia. In combination with other agents MET is an important treatment agent for the eradication of *Helicobacter pylori*, and is recommended in the management of diarrhea caused by *Clostridium difficile* and bacterial vaginosis. Although MET has been used for more than 45 years, the frequency of resistance among Gram-negative rods is still quite low. However, decreased susceptibility in the *Bacteroides* species, as well as metronidazole-resistant *Helicobacter pylori* strains have been reported. Metronidazole does not act against aerotolerant anaerobes e.g. *Propionibacterium* spp. and *Actinomyces* spp. and is ineffective against aerobic bacteria [31]. Moreover, unsuccessful therapies using this antibiotic are observed with increasing frequency [32] in *C. difficile* infections, where the pathophysiology is associated with two enterotoxins, A and B, that cause inflammatory changes in the mucosa [33]. Our results of the killing assay show an enhanced (better than LL-37 and metronidazole) activity of both ceragenins against clinical, toxinogenic *C. difficile* strain*.* Strains tested in another study displayed various responses to cathelicidin LL-37. Epidemic-associated 027 ribotype isolates tended to be more resistant than others [34]. Hing et al. have shown, both in vitro and in vivo*,* comparable results demonstrating the mild activity of LL-37 and also no significant antibacterial effects of mouse cathelicidin (mCRAMP) against *C. difficile* [35]. Interestingly, Oh et al. showed that exogenous intracolonic administration of cathelicidin significantly reduced toxin A-associated intestinal inflammation and colonic tissue damage by inhibiting tissue myeloperoxidase activity, macrophage infiltration, apoptosis and tumor necrosis factor α (TNFα) levels. The authors postulated that the potential value of these compounds is due to their anti-inflammatory effects against the actions of toxins in the intestinal mucosa and that the exogenous administration of cathelicidin could be a new anti-inflammatory treatment for *Clostridium difficile* infections. In *Clostridium difficile* infections the stability of the antimicrobial in feces is crucial. Using an experimental fecal model it was observed that the D-isomer of temporin B were inactivated more slowly in the fecal milieu than the L-isomer. Moreover, D-isomers were more active than L forms [25]. It is also known that cathelicidin LL-37 and other antibacterial peptides are not stable in body fluids and their non-peptide mimics seem to be better candidates as future antibacterial drugs [36]. It might be also assumed that ceragenins stability in feces will be satisfactory. While antibiotic resistance is currently not a major concern for most anaerobic bacteria, decreasing efficacy of several classes of antimicrobials have been observed, particularly in clinical situations where antibiotics are used for extended periods of time, such as acne vulgaris.

*Propionibacterium acnes* is resistant to commonly used antibiotics including erythromycin, clindamycin and tetracyclin, which makes the treatment of acne challenging. Antibiotic resistance is due in part to insufficient antibiotic penetration into the sebaceous hydrophobic environment of the skin where *P. acnes* lives and also low, ineffective antimicrobial doses [37]. Our experiments have shown clinically relevant antibacterial activity of LL-37 peptide and CSAs against *P*. *acnes*. Several other CAPs have been found to exert anti*-P. acnes* properties including the cecropin-melittin analogues, temporins [25, 26]. Snake cathelicidin-BF displayed not only significant antimicrobial activity against *P.acne*s but also inhibitory effects on cytokine secretion induced by this bacterium and no detrimental influence on skin cells [38]. Beneficial features of CAPs and their derivatives were used in engineering the peptide-aminoglycoside polycationic hybrid – Pentobra, which has multiple mechanisms of antibacterial action and the potential for effective treatment in cutaneous environments. This molecule is a combination of tobramycin that has ribosomal activity and a short 12 amino acid peptide with activity against bacterial membranes. This composition has strong bactericidal activity (much better than free tobramycin which in fact does not act against anaerobic bacteria), potential anti-inflammatory effects (the suppression of *P. acnes*-induced chemokines) and in vitro activity in comedone extracts isolated from human donors. Such an approach - a combination of two substances with distinct mechanisms of action - demonstrates the new therapeutic strategy enhancing the effectiveness of antimicrobial action and minimizing chances of bacterial resistance, which is important in long topical application [37]. The results of our study have displayed excellent activities of ceragenins against *P. acnes*. CSA-13 and CSA-131 were more effective (including at lower concentrations) than the LL-37 peptide. Apart from their enhanced bactericidal activity, the ceragenins showed also a good penetration into the lipid membrane. Moreover, ceragenins as amphiphilic substances, with their mode of action and low risk to develop resistance, appear to be promising candidates for effective, long topical application and treatment in diseases such as acne and biomaterials – related infections with delayed presentation after spinal instrumentation, shunting for hydrocephalus*,* arthroplasty that can be also caused by *P. acnes*. A key factor in these infections is biofilm formation. It has been shown in vitro, that conventional antibiotics such as penicillin or linezolid plus rifampicin are effective against *P. acnes* biofilms [39]. Our investigation using ampicillin confirms effective activity of antibiotics from the penicillin group against *P. acnes* biofilm even the methods of our experiment differ from those use previously [39]. The effect of ceragenin CSA-131 is comparable to ampicillin, but the influence of cathelicidin LL-37 is slightly weaker. Because, other anaerobes, especially belonging to the genera *Bacteroides*, *Clostridium*, *Fusobacterium*, *Finegoldia* (formerly Peptostreptococcus), *Prevotella*, and *Veillonella* have the ability to develop biofilms [40], the antibiofilm activity is another advantage of ceragenins as potential antimicrobial drugs. According to our results, ceragenin CSA-131 significantly inhibited biofilms formed by *B. fragilis.* The CSA-13 and LL-37 antibiofilm activity were comparable to metronidazole. In general, we have observed heightened antimicrobial activity for ceragenins compared to LL-37 against all tested anaerobic bacteria (planktonic forms as well as biofilm forms). The advantage of ceragenins over cathelicidin is in agreement with previous observations involving aerobic bacteria [41] and fungi [42]. Previous studies suggest that the anti-inflammatory effects of CSAs in conjunction with their wide antimicrobial activities indicate their potential use as novel therapeutic agents for the topical treatment of diseases where both bacterial as well as persistent inflammatory components are involved eg. acne vulgaris, periodontal diseases. Despite the benefits of ceragenins, their potential for systemic use is limited by their unsatisfactory safety profile, particularly the possibility for red blood cells (RBCs) hemolysis, which occurs in higher concentrations [43]. These potential harmful effects can be reduced by the use of nanoparticles. Nanoparticles can serve as drug delivery systems [15] and can enhance antibacterial activity. Previous studies with *Pseudomonas aeruginosa* have shown that immobilization of ceragenin on the nanoparticle surface significantly reduced membrane toxicity of CSA-13 to red blood cells. Moreover, MNP@CSA-13 was efficient in killing bacteria and preventing biofilm formation. Uncoated MNPs also displayed antibacterial activity against this bacterium [19]. As far as we know prior to this paper, no data showing the influence of MNPs on anaerobic bacteria have been published. In the current study, we assessed the activity of ceragenins immobilized on MNPs against selected anaerobic bacteria. MIC/MBC values for all of the tested strains using nanoparticles coated with metronidazole, LL-37, CSA-13 and CSA-131 were comparable to those when antimicrobials were used alone. Such data suggest that the tested antimicrobials, covalently attached to nanoparticles retain bactericidal activity against anaerobic bacteria. This is in the accordance with data from aerobic bacteria and fungi [44]. But, in our study with anaerobic bacteria MIC/MBC values for MNPs alone were very high. Surprisingly, in the killing assay nanoparticles without antimicrobials exerted very high antibacterial activity, even better than MNPs attached to antimicrobials. As previously mentioned, different experimental conditions in the evaluation of MIC/MBC values and killing assay assessment, particularly the different incubation times (1 h versus 48-72 h) might greatly influence the results and may indicate that MNPs do not act in real anaerobic conditions which are not achieved during 1 h incubation. Another unexpected finding is that ceragenins and LL-37 without nanoparticles acted slightly better then in combinations with nanoparticles. The opposite effect was observed for metronidazole as well. It is probable that, the usage of one amine group from ceragenin to decorate the nanoparticle surface slightly diminishes the antibacterial activity of tested agents. Evaluation of the antibiofilm activity showed that most antimicrobials attached to MNPs, even MNPs alone exhibited antibiofilm activity. CSA-131, when used alone, exerted the strongest antibiofilm activity among all tested compounds. Our study demonstrates that interpreting the results from evaluation of ceragenins activity against anaerobic bacteria is challenging. Anaerobic bacteria are difficult to culture and many factors involved in culture can influence the results: medium, time, the method of assessment the antibacterial activity, the way of reading results etc. Although, the MIC/MBC micodilution test remains the standard method for the in vitro evaluation of antimicrobial activity, CLSI does not recommend it for all anaerobic bacteria. As we have investigated this method needs some modification when activity of CAPs and their mimics are assessed so comparing results from different study are difficult because of various conditions used in experiments. In fact, in our opinion not the real MIC/MBC values but only trends can be comparable even when reference strains from ATCC collection are investigated.

## Conclusions

Our preliminary data indicate that the antibacterial spectrum of ceragenins should be expanded to include selected anaerobic bacteria causing human infections with high frequency such as: *Bacteroides*, *Prevotella*, *Clostidum*, *Propionibacterium* species. Broad antimicrobial spectrum and antibiofilm activity are promising advantages of these compounds as potential therapeutics especially in treatment of mixed, polymicrobial infections, when the utility of one antimicrobial with wide spectrum is necessary. The potential application of ceragenins as new antibacterials needs further investigations. It would be worth to assess the ceragenin activity against additional bacteria responsible for periodontal disease, bacterial vaginosis and other very frequent anaerobic or mixed infections, as well as their stability in side of infections, pharmacokinetic and pharmacodynamic properties. The poor activity of nanoparticles against anaerobic bacteria do not exclude its potential usage in treatment of infections where anaerobic bacteria are involved. The advantage would be the utility of nanoparticles as drug delivery systems, which is very important when the effective concentration of antimicrobials in sites of infection is not easy to achieve. The utility of nanoparticles could be also an interesting solution for systemic applications. Obviously, the influence of magnetic nanoparticles to human cells as well as to natural microbiota has to be thoroughly examined.

## Abbreviations

AM: Ampicillin
CAMEL: Hybrid peptide cecropin-melittin analogues
CAPs: Cationic antimicrobial peptides
CFU: Colony forming unit
CLSI: Clinical Laboratory Standards Institute
CSA-13: Ceragenin CSA-13
CSA-131: Ceragenin CSA-131
DDS: Drug delivery system
LL-37: Cathelicidin delivered 37 amino acid peptide
LPS: Lipopolysaccharide
MBC: Minimum bactericidal concentration
mCRAMP: Mouse cathelicidin
MET: Metronidazole
MIC: Minimum inhibitory concentration
MNP: Magnetic nanoparticles
MNP@CSA-13: CSA-13 attached to magnetic nanoparticles
MNP@CSA-131: CSA-131 attached to magnetic nanoparticles
MNP@LL37: LL-37 attached to magnetic nanoparticles
MNP@MET: Metronidazole attached to magnetic nanoparticles
OD: Optical density
PBS: Phosphate-buffered saline
SMAP-29: Sheep myeloid antimicrobial peptide 29
TNFα: Tumor necrosis factor α

## References

[1] Sekirov I, Russell SL, Antunes LC, Finlay BB (2010). Gut microbiota in health and disease. Physiol Rev.

[2] Park Y, Choi JY, Yong D, Lee K, Kim JM (2009). Clinical features and prognostic factors of anaerobic infections: a 7-year retrospective study. Korean J Intern Med.

[3] Brook I (2010). The role of anaerobic bacteria in bacteremia. Anaerobe.

[4] Lee DG (2009). Clinical significance of anaerobic infections. Korean J Intern Med.

[5] Ali Mohammed MM, Nerland AH, Al-Haroni M, Bakken V. Characterization of extracellular polymeric matrix, and treatment of Fusobacterium nucleatum and Porphyromonas gingivalis biofilms with DNase I and proteinase K. J Oral Microbiol. 2013;5 10.3402/jom.v5i0.20015 PMC3559756 23372876

[6] Machado D, Castro J, Palmeira-de-Oliveira A, Martinez-de-Oliveira J, Cerca N (2015). Bacterial Vaginosis biofilms: challenges to current therapies and emerging solutions. Front Microbiol.

[7] Vaishnavi C (2010). Clinical spectrum & pathogenesis of Clostridium Difficile associated diseases. Indian J Med Res.

[8] Nord CE, Oprica C (2006). Antibiotic resistance in Propionibacterium acnes. Microbiological and clinical aspects. Anaerobe.

[9] Shindo Y, Dobashi Y, Sakai T, Monma C, Miyatani H, Yoshida Y (2015). Epidemiological and pathobiological profiles of Clostridium Perfringens infections: review of consecutive series of 33 cases over a 13-year period. Int J Clin Exp Pathol.

[10] Feleke G, Forlenza S (1991). Anaerobic infections. The basics for primary care physicians. Postgrad Med.

[11] Akhi MT, Ghotaslou R, Beheshtirouy S, Asgharzadeh M, Pirzadeh T, Asghari B, Alizadeh N, Toloue Ostadgavahi A, Sorayaei Somesaraei V, Memar MY (2015). Antibiotic susceptibility pattern of aerobic and anaerobic bacteria isolated from surgical site infection of hospitalized patients. Jundishapur J Microbiol.

[12] Durr UH, Sudheendra US, Ramamoorthy A (2006). LL-37, the only human member of the cathelicidin family of antimicrobial peptides. Biochim Biophys Acta.

[13] Epand RF, Pollard JE, Wright JO, Savage PB, Epand RM (2010). Depolarization, bacterial membrane composition, and the antimicrobial action of ceragenins. Antimicrob Agents Chemother.

[14] Pollard J, Wright J, Feng Y, Dianliang G, Genberg C, Savage PB (2009). Activities of Ceragenin CSA-13 against established biofilms in an in vitro model of catheter decolonization. Antiinfect Agents Med Chem.

[15] Wilczewska AZ, Niemirowicz K, Markiewicz KH, Car H (2012). Nanoparticles as drug delivery systems. Pharmacol Rep.

[16] Niemirowicz K, Swiecicka I, Wilczewska AZ, Misztalewska I, Kalska-Szostko B, Bienias K, Bucki R, Car H (2014). Gold-functionalized magnetic nanoparticles restrict growth of Pseudomonas Aeruginosa. Int J Nanomedicine.

[17] Ding B, Guan Q, Walsh JP, Boswell JS, Winter TW, Winter ES, Boyd SS, Li C, Savage PB (2002). Correlation of the antibacterial activities of cationic peptide antibiotics and cationic steroid antibiotics. J Med Chem.

[18] Massart R (1981). Preparation of aqueous magnetic liquids in alkaline and acidic media. IEEE Trans Magn.

[19] Niemirowicz K, Surel U, Wilczewska AZ, Mystkowska J, Piktel E, Gu X, Namiot Z, Kulakowska A, Savage PB, Bucki R (2015). Bactericidal activity and biocompatibility of ceragenin-coated magnetic nanoparticles. J Nanobiotechnol.

[20] CLSI (2012). Methods for antimicrobial susceptibility testing of anaerobic bacteria. Approved standard, 8th ed CLSI document M11-A8.

[21] Bucki R, Niemirowicz K, Wnorowska U, Watek M, Byfield FJ, Cruz K, Wroblewska M, Janmey PA (2015). Polyelectrolyte-mediated increase of biofilm mass formation. BMC Microbiol.

[22] Brook I (2011). Antimicrobial treatment of anaerobic infections. Expert Opin Pharmacother.

[23] Chin JN, Rybak MJ, Cheung CM, Savage PB (2007). Antimicrobial activities of ceragenins against clinical isolates of resistant Staphylococcus Aureus. Antimicrob Agents Chemother.

[24] Vila-Farres X, Callarisa AE, Gu X, Savage PB, Giralt E, Vila J (2015). CSA-131, a ceragenin active against colistin-resistant Acinetobacter Baumannii and Pseudomonas Aeruginosa clinical isolates. Int J Antimicrob Agents.

[25] Oh H, Hedberg M, Wade D, Edlund C (2000). Activities of synthetic hybrid peptides against anaerobic bacteria: aspects of methodology and stability. Antimicrob Agents Chemother.

[26] Urban E, Nagy E, Pal T, Sonnevend A, Conlon JM (2007). Activities of four frog skin-derived antimicrobial peptides (temporin-1DRa, temporin-1Va and the melittin-related peptides AR-23 and RV-23) against anaerobic bacteria. Int J Antimicrob Agents.

[27] Arzese A, Skerlavaj B, Tomasinsig L, Gennaro R, Zanetti M (2003). Antimicrobial activity of SMAP-29 against the Bacteroides Fragilis group and clostridia. J Antimicrob Chemother.

[28] Bachrach G, Altman H, Kolenbrander PE, Chalmers NI, Gabai-Gutner M, Mor A, Friedman M, Steinberg D (2008). Resistance of Porphyromonas gingivalis ATCC 33277 to direct killing by antimicrobial peptides is protease independent. Antimicrob Agents Chemother.

[29] Ouhara K, Komatsuzawa H, Yamada S, Shiba H, Fujiwara T, Ohara M, Sayama K, Hashimoto K, Kurihara H, Sugai M (2005). Susceptibilities of periodontopathogenic and cariogenic bacteria to antibacterial peptides, {beta}-defensins and LL37, produced by human epithelial cells. J Antimicrob Chemother.

[30] Epand RF, Savage PB, Epand RM (2007). Bacterial lipid composition and the antimicrobial efficacy of cationic steroid compounds (Ceragenins). Biochim Biophys Acta.

[31] Lofmark S, Edlund C, Nord CE (2010). Metronidazole is still the drug of choice for treatment of anaerobic infections. Clin Infect Dis.

[32] Vincent Y, Manji A, Gregory-Miller K, Lee C (2015). A review of Management of Clostridium difficile infection: primary and recurrence. Antibiotics (Basel).

[33] Rineh A, Kelso MJ, Vatansever F, Tegos GP, Hamblin MR (2014). Clostridium Difficile infection: molecular pathogenesis and novel therapeutics. Expert Rev Anti-Infect Ther.

[34] McQuade R, Roxas B, Viswanathan VK, Vedantam G (2012). Clostridium Difficile clinical isolates exhibit variable susceptibility and proteome alterations upon exposure to mammalian cationic antimicrobial peptides. Anaerobe.

[35] Hing TC, Ho S, Shih DQ, Ichikawa R, Cheng M, Chen J, Chen X, Law I, Najarian R, Kelly CP (2013). The antimicrobial peptide cathelicidin modulates Clostridium Difficile-associated colitis and toxin A-mediated enteritis in mice. Gut.

[36] Bucki R, Namiot DB, Namiot Z, Savage PB, Janmey PA (2008). Salivary mucins inhibit antibacterial activity of the cathelicidin-derived LL-37 peptide but not the cationic steroid CSA-13. J Antimicrob Chemother.

[37] Schmidt NW, Agak GW, Deshayes S, Yu Y, Blacker A, Champer J, Xian W, Kasko AM, Kim J, Wong GC (2015). Pentobra: a potent antibiotic with multiple layers of selective antimicrobial mechanisms against Propionibacterium acnes. J Invest Dermatol.

[38] Wang Y, Zhang Z, Chen L, Guang H, Li Z, Yang H, Li J, You D, Yu H, Lai R (2011). Cathelicidin-BF, a snake cathelicidin-derived antimicrobial peptide, could be an excellent therapeutic agent for acne vulgaris. PLoS One.

[39] Bayston R, Nuradeen B, Ashraf W, Freeman BJ (2007). Antibiotics for the eradication of Propionibacterium acnes biofilms in surgical infection. J Antimicrob Chemother.

[40] Donelli G, Vuotto C, Cardines R, Mastrantonio P (2012). Biofilm-growing intestinal anaerobic bacteria. FEMS Immunol Med Microbiol.

[41] Wnorowska U, Niemirowicz K, Myint M, Diamond SL, Wroblewska M, Savage PB, Janmey PA, Bucki R (2015). Bactericidal activities of cathelicidin LL-37 and select cationic lipids against the hypervirulent Pseudomonas Aeruginosa strain LESB58. Antimicrob Agents Chemother.

[42] Durnas B, Wnorowska U, Pogoda K, Deptula P, Watek M, Piktel E, Gluszek S, Gu X, Savage PB, Niemirowicz K (2016). Candidacidal activity of selected Ceragenins and human cathelicidin LL-37 in experimental settings mimicking infection sites. PLoS One.

[43] Lai XZ, Feng Y, Pollard J, Chin JN, Rybak MJ, Bucki R, Epand RF, Epand RM, Savage PB (2008). Ceragenins: cholic acid-based mimics of antimicrobial peptides. Acc Chem Res.

[44] Niemirowicz K, Swiecicka I, Wilczewska AZ, Markiewicz KH, Surel U, Kulakowska A, Namiot Z, Szynaka B, Bucki R, Car H (2015). Growth arrest and rapid capture of select pathogens following magnetic nanoparticle treatment. Colloids Surf B Biointerfaces.

